# Prevalence and determinants of pre-lacteal feeding in South Sudan: a community-based survey

**DOI:** 10.1080/16549716.2018.1523304

**Published:** 2018-10-08

**Authors:** Justin Bruno Tongun, Mohamedi Boy Sebit, Grace Ndeezi, David Mukunya, Thorkild Tylleskar, James K. Tumwine

**Affiliations:** a Centre for International Health, University of Bergen, Bergen, Norway; b Department of Paediatrics and Child Health, College of Medicine, University of Juba, Juba, South Sudan; c Department of Internal Medicine, College of Medicine, University of Juba, Juba, South Sudan; d Department of Paediatrics and Child Health, School of Medicine, College of Health Sciences, Makerere University, Kampala, Uganda

**Keywords:** pre-lacteal feeding (PLF), predictors, infant, newborn, conflict

## Abstract

**Background**: Pre-lacteal feeding (PLF) is a barrier to optimal breastfeeding and increases the risk of diarrhoea and acute respiratory tract infections in infants.The prevalence and predictors of PLF are not well studied in South Sudan. Understanding the predictors of PLF is crucial in designing interventions to increase exclusive breastfeeding (EBF) rates.

**Objective**: To assess the prevalence and factors associated with PLF in Jubek State, South Sudan.

**Method**: This was a community based cross-sectional study of 810 mothers of children under two years of age in Jubek State, South Sudan. Mothers were interviewed in their homes using a semi-structured questionnaire to collect data on PLF, socio-demographic and birth characteristics. Multivariable analysis was used to identify factors independently associated with PLF.

**Results**: A total of 426/810 (53 %), 95% confidence interval (CI) [48 %, 59 %] mothers had given pre-lacteal feeds to their infants. The commonest pre-lacteal feeds included glucose solution (54%), water (26%), and infant formula (14%). Having received antenatal breastfeeding counselling decreased the odds of PLF [adjusted odds ratio (AOR) 0.60; 95% CI (0.43, 0.82)]; while discarding of colostrum increased the use of pre-lacteal feeds [AOR 1.57; 95% CI (1.17, 2.11)].

**Conclusion**: The prevalence of PLF in South Sudan is high. Predictors of PLF included lack of breastfeeding counselling and discarding of colostrum. Infant feeding counselling should be given to all pregnant women in the health facilities and communities. The counselling should emphasize the health benefits of colostrum and discourage the practice of discarding it.

## Background

The United Nations Sustainable Development Goals recommended a further reduction of neonatal and under-fives’ mortality by 2030 [1]. Optimal breastfeeding is one of the interventions that improve child survival [2]. It has three elements according to the World Health Organization (WHO) namely: early initiation of breastfeeding (within one hour of birth); exclusive breastfeeding (EBF) – breastmilk with no other foods or liquids – in the first six months of life; and continued breastfeeding up to two years of age and beyond, while receiving appropriate complementary foods [3].

**Figure 1.**
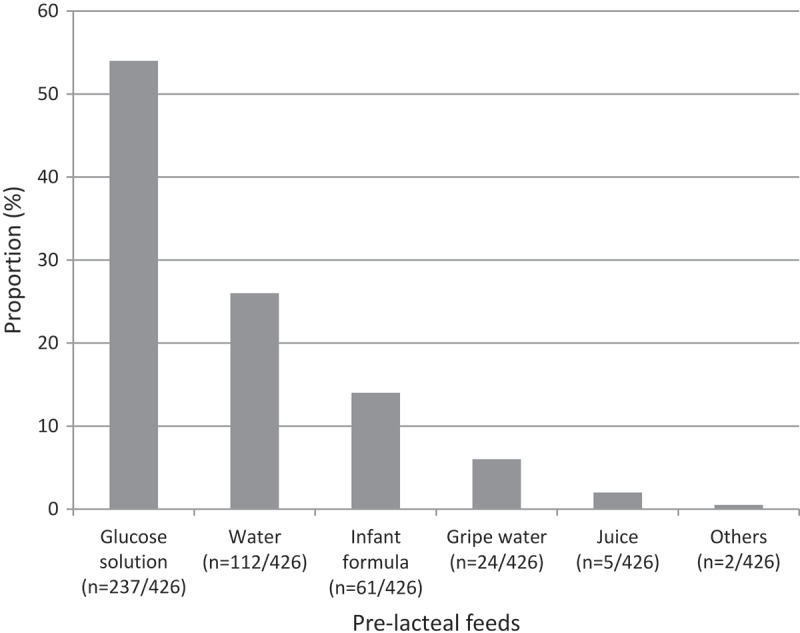
Types of pre-lacteal feeds used among mothers of children aged less than two years surveyed in South Sudan.

*The Lancet* series on breastfeeding estimated that, globally, optimal breastfeeding may save the lives of 823,000 children aged less than five-years-old annually, equivalent to 14% of the child deaths [4]. A multi-country study with close to 100 000 mother–infant pairs found that both early initiation and EBF were independently associated with a lower risk of child death [5]. Pre-lacteal feeding (PLF) is defined as giving newborns liquids or foods other than breastmilk before breastfeeding is established. It is one of the practices jeopardizing optimal breastfeeding in low and middle-income countries (LMIC) [6,7]. The Baby Friendly Hospital Initiative (BFHI), in its 10 steps to successful breastfeeding, discourages this practice [8]. The rationale behind discouraging PLF is that the feeds are easily contaminated. This might introduce harmful micro-organisms that increase the risk of diarrhoea and disrupt establishment of normal flora in the infant’s gastrointestinal tract [9]. PLF has, also, been associated with a fourfold increased risk in infant deaths from infectious diseases [10]. Several studies have found that socio-economic conditions, lack of knowledge and cultural beliefs may contribute to PLF [11–13].

South Sudan, the world’s youngest country, gained independence in 2011 [14]. It has been plagued by conflict and widespread population displacement with nearly 5 million people in need of humanitarian aid [15]. The civil unrest resulted in displacement that negatively influences infant feeding practices [16]. UNICEF and non-government organizations (NGOs) estimates that 45% of children below six-months-old are exclusively breastfed [17] and pre-lacteal feeds such as water and cows’ milk are commonly used [18]. However, published information on predictors of PLF in South Sudan is lacking. Understanding the predictors of PLF is critical in developing strategies to mitigate this practice. This study describes the prevalence and factors associated with PLF in South Sudan.

## Methods

### Study design, setting and participants

This was a community based cross-sectional study conducted among mothers of children aged less than two years.

The study was carried out in Jubek State, South Sudan, from October to December 2016. Jubek State has an estimated population of 530,000 most of whom live in rural areas [19]. It has 12 counties and one city council (Juba). The study was done in the four counties of Ladu (N = 24,000), Luri (30,744), Mangala (9,631) and Rajaf (21,000) [19].

All consenting mothers who had a child aged less than two years were included in the study. This included mother–infant pairs who had been present in the village at least 24 hours before the survey. For mothers with more than one eligible child, the youngest was selected.

### Sample size estimation

The sample size estimation was based on the two objectives. For the prevalence of PLF, the calculated sample size was 810 mother–infant pairs. This sample size was obtained using Open-Epi software [20]. The following considerations were made during the calculation: 43% prevalence of PLF from a study in Uganda [21]; precision of 5%; design effect of 2 and 7.5% non-response.

We also calculated the sample size for factors associated with PLF using Open-Epi for detecting variations between proportions of two groups (Kelsey formula) [20]. In this calculation, the place of birth was used to calculate the sample size required to detect differences in the proportion of PLF between mothers who had given birth at home and those who had hospital births. Using a study in South Sudan where 87% of mothers gave birth at home [22], we assumed that the prevalence of PLF was 70% among mothers who had given birth at home, and 48% for those delivering in hospital. This generated a sample size of 349 for the objective on factors associated with PLF. Therefore, we took the larger sample size of 810 since this would give adequate sample size for each objective.

### Sampling procedure

We utilized a modified two stage cluster design survey method developed by the Expanded Program of Immunization of the WHO [23]. In the first stage, all the clusters (43 villages) in the four counties were recorded with their populations. Using probability proportional to size, we selected 30 villages. In the second stage, we asked the village leaders to enumerate and make a list of all households, since there was no list of households available in South Sudan. In each village, we used the generated household list to select an index house by simple random sampling. After choosing the first house, the next house was the one with the door facing and closest to the preceding house. One mother who had an infant aged 0–23 months was interviewed in each house. This procedure was repeated until 27 mother–infant pairs had been recruited in each village.

Eight trained research assistants conversant with the study area and fluent in *Bari* (the local language) collected the data. The interviews were done in a private area in the mother’s home, away from the other members of the family.

### Study variables

The outcome variable was PLF. Mothers were asked: ‘Did you give [NAME] anything else in the first three days of life apart from breast milk?’ If yes, they were asked: ‘what was [NAME] given to drink?’ (The pre-coded options were: water; glucose solution, non-human milk; formula milk; juice; gripe water; solid food and others). The independent variables were maternal age at the last birth, place of residence categorized as ‘rural’ or ‘urban’, marital status categorized as ‘married’ or ‘single’ (single, separated, divorced, widowed), maternal education was categorized as ‘none’ (no formal education), ‘primary’, and ‘≥ secondary’, maternal employment (any job outside the home) was categorized as ‘employed’ and ‘unemployed’, child’s sex was categorized as ‘male’ or ‘female’, antenatal care visit was categorized as ‘0–3ʹ, ‘≥ 4ʹ, place of birth was categorized as ‘health facility’ or ‘home’, mode of birth was categorized as ‘normal birth’ or ‘caesarean section’, type of birth was categorized as ‘single’ or ‘multiple’, birth order was categorized as ‘primipara’ or ‘multipara’, discarding of colostrum (expressing and throwing away of the first milk) was categorized as ‘yes’ and ‘no’, exposure to infant formula advertisement a month before birth was categorized as ‘yes’ and ‘no’, antenatal breastfeeding counselling was categorized as ‘yes’ and ‘no’, supported/assisted to breastfeed was categorized as ‘yes’ and ‘no’, and house ownership categorized as ‘yes’ and ‘no’.

### Data quality and analysis

We used a study tool developed from the WHO guidelines on infant and young child feeding (IYCF) [24] and a study on early initiation of breastfeeding and EBF in Uganda [25]. The questionnaire was translated into the local language *Bari*, and translated back into English, by a different expert. Pilot testing was conducted to test the feasibility of study questionnaire. The principal investigator (JBT) checked the data daily, for completeness and consistency. Data was cleaned, coded and double entered into Epi Info version 6 and stored in a secure password-protected computer.

Continuous descriptive variables were reported as means and standard deviations and the categorical variables as proportions. Chi square tests were performed to identify independent variables at bivariable analysis that were associated with outcome variables. Predictors of PLF from the literature and those with a *p-*value ≤0.25, not in the causal pathway and not strongly collinear with other independent variables were entered into the initial multivariable logistic regression model. We evaluated for collinearity and predictors with variance inflation factor >10 were considered strongly collinear. In case of collinearity, the predictor with a stronger measure of association with the outcome variable was retained and the other dropped from the model. We formed interaction terms for variables that were significantly associated with pre lacteal feeding, after running a backward stepwise model. We assessed for interaction by comparing the original model with the model with interaction terms using a chunk test. In the final model building we included statistically significant variables in the original model (*p *< 0.05), significant variables from the literature, and confounders in the model. Any variable that caused a difference of ≥10% between the crude and adjusted measures of association of any of the variables in the model was maintained in the model as a confounder. Independent variables with a *p*-value of less than 0.05 were considered significant. Discarding of colostrum could be a consequence and not a cause of the PLF. To control this situation, we repeated the analysis without this variable.This study used STATA version 14 (STATA Corp LLC, Texas, USA) for data analysis.

## Results

A total of 810 mother–infant pairs were included in the study, as shown in Tables 1 and 2. The mean age of the mothers was 26.6 years, with a standard deviation (SD) of 5.5. The mean (SD) age of the children was 12.4 (6.9) months. Most mothers were married, over half had no formal education, and only a quarter gave birth at a health facility.

**Table 1.** Baseline characteristics and PLF among mothers of children aged less than two years surveyed in South Sudan.

Characteristics	All participants	Pre-lacteal feeding
	N = 810	N = 426
	n (%)	n (%)
Mother’s age		
≤19	59 (7.3)	33 (7.7)
20–24	205 (25.3)	100 (23.5)
25–29	289 (35.7)	161 (37.8)
30–34	178 (22.0)	93 (21.8)
≥35	79 (9.7)	39 (9.2)
Marital status		
Married	793 (97.9)	415 (97.4)
Single	17 (2.1)	11 (2.6)
Mother’s education		
None	516 (63.7)	280 (65.7)
Primary	228 (28.2)	120 (28.2)
≥Secondary	66 (8.1)	26 (6.1)
Mother’s employment		
Employed	120 (14.8)	57 (13.4)
Unemployed	690 (85.2)	369 (86.6)
House ownership		
Yes	790 (97.5)	417 (9.0)
No	20 (2.5)	251 (2.1)
Child’s sex		
Male	390 (48.2)	230 (54.0)
Female	420 (51.9)	196 (46.0)

**Table 2.** Birth characteristics and PLF among mothers of children aged less than twoyears surveyed in South Sudan.

Characteristics	All participants	Pre-lacteal feeding
	N = 810	N = 426
	n (%)	n (%)
Antenatal care visits		
Four or more	591 (73.0)	299 (70.2)
0–3	219 (27.0)	127 (29.8)
Place of birth		
Health facility	209 (25.8)	103 (24.2)
Home	601 (74.2)	323 (75.8)
Mode of child birth		
Normal birth	793 (97.9)	414 (97.2)
Caesarean section	17 (2.1)	12 (2.8)
Type of birth		
Single	789 (97.4)	416 (97.7)
Multiple	21 (2.6)	10 (2.4)
Birth order		
Primipara	138 (17.0)	74 (17.4)
Multipara	672 (83.0)	352 (82.6)
Breastfeeding counselling		
No	508 (62.7)	251 (58.9)
Yes	302 (37.3)	175 (41.1)
Breastfeeding support		
No	641 (79.1)	344 (80.8)
Yes	169 (20.9)	82 (19.3)
Discarding of colostrum		
No	496 (61.2)	239 (56.1)
Yes	314 (38.8)	187 (43.9)
Exposed to infant formula advertisement		
No	563 (69.5)	350 (82.2)
Yes	247 (30.5)	76 (17.8)

Fifty-three per cent (426/810), 95% CI [48 %, 59 %] of the mothers had given pre-lacteal feeds, as shown in Tables 1 and 2. The common pre-lacteal feeds included glucose solution, plain water, infant formula, gripe water, and fruit juice, as shown in Figure 1.

In the bivariable analysis, the factors associated with PLF were maternal education, caesarean section, having received antenatal breastfeeding counselling and discarding of colostrum, as shown in Table 3. In the multivariable analysis, having received antenatal breastfeeding counselling decreased PLF [AOR 0.60; 95% CI (0.43, 0.82)]; while discarding of colostrum increased it [AOR 1.57; 95% CI (1.17, 2.11)], as shown in Table 3.

**Table 3.** Bivariable and multivariable analysis of the use of PLF among mothers of children aged less than two years surveyed in South Sudan.

Characteristic	Bivariable	Multivariable model 1	Multivariable model 2
	N = 810	N = 810	N = 810
	OR (95%CI)	Adjusted odd ratio AOR (95%CI)	Adjusted odd ratio AOR (95%CI)
Mother’s age			
≤19	1.5 (0.79, 2.82)	-	-
20–24	1		
25–29	1.2 (0.86, 1.76)		-
30–34	1.1 (0.66, 1.75)	-	-
≥35	1.1 (0.60, 1.88)	-	-
Marital status			
Married	1		
Single	1.67 (0.46,6.03)	-	-
Mother education			
No formal education	1	1	1
Primary	0.94 (0.62,1.42)	0.63 (0.33, 1.19)	0.60 (0.31,1.09)
≥Secondary	0.55 (0.31,0.97)	0.76 (0.24, 1.26)	0.66 (0.21,2.04)
Mother employment			
Employed	1	1	1
Unemployed	0.79 (0.49,1.26)	0.84 (0.56, 1.26)	0.85 (0.57,1.28)
House ownership			
Yes	1		
No	0.73 (0.24,2.20)	-	-
Child sex			
Male	1		
Female	1.20 (0.89,1.61)	-	-
Antenatal care visits			
Four or more	1		
0–3	0.85 (0.65,1.13)	-	-
Place of birth			
Health facility	1	1	1
Home	1.20 (0.81,1.77)	1.23 (0.84,1.81)	1.36 (0.92,2.01)
Mode of birth			
Normal birth	1		
Caesarean section	2.20 (0.87,5.58)	-	-
Type of birth			
Single	1		
Multiple	0.82 (0.27,2.48)	-	-
Birth order			
Primipara	1		
Multipara	0.95 (0.70,1.29)	-	-
Breastfeeding counselling			
No	1	1	1
Yes	0.71(0.50,1.08)	0.60 (0.43,0.82)	0.63 (0.46,0.87)
Breastfeeding support			
Yes	1		
No	1.22 (0.81,1.86)		
Discarding of colostrum			
No	1	1	
Yes	1.58 (1.11,2.26)	1.57 (1.17, 2.11)	-
Exposure to infant formula advertisement			
No	1		
Yes	1.05 (0.72,1.51)	-	-

Multivariable model 1 including all the predefined variables.

Multivariable model 2 as in number 1 but excluding the discarding of colostrum.

## Discussion

In this study, more than half (53%) the mothers practised PLF. This finding is not surprising, since it is consistent with studies in the East African region. For example, a report from Eastern Uganda by Engebretsen et.al [26], found that 57% of mothers had given pre-lacteal feeds. Nevertheless, a lower prevalence of PLF was reported from the Kilimanjaro region in northern Tanzania (1%) [27], and from northwest (26.8%) [28] and south Ethiopia(25.5%) [7]. Whereas some low income countries have extension community health workers who move among the villages promoting recommended maternal and newborn care practices [7], such programs are non-existent in South Sudan. The lack of organized programs that specifically focus on promotion of breastfeeding might explain the high prevalence of PLF in our study. A much higher prevalence of PLF was reported in North West Nigeria (85%) [29]. The differences in PLF rates might be partly attributed to variations in study participants and cultural beliefs associated with the practice.

The commonest pre-lacteal feeds in this study were sugar solution (56%), plain water (26%) and infant formula (14%). This agrees with previous studies in Egypt and Kenya [30,31] where similar pre-lacteal feeds were used. Another study in northwest Nigeria reported cows’ milk as the most preferred pre-lacteal feed [29]. Participants in the current study were largely peasant farmers with less access to cows’ milk and were more likely to give sugar solution, because sugar was readily available. In addition, the use of sugar solution and infant formula was partly due to maternal misconception that the infant might be hungry or hypoglycaemic, especially before lactation is fully established. On the other hand, mothers who gave plain water thought that the infant might be thirsty because of the hot climate in South Sudan.

Mothers who discarded colostrum were two times more likely to practise PLF compared to those who did not. Most probably, the mothers who discarded colostrum were left with no alternative but to give pre-lacteal feeds. Studies from Egypt, Ethiopia and Nepal have also reported that discarding of colostrum was associated with PLF [30,32–34]. A report from Raya Kobo district, Ethiopia [35] found that untrained traditional birth attendants and family members encouraged mothers to discard colostrum and practise PLF [35]. Our study did not qualitatively probe the reasons for discarding colostrum and the role of cultural beliefs on PLF.

We found mothers who received breastfeeding counselling during antenatal care were less likely to give pre-lacteal feeds compared to those who were not counselled on breastfeeding. Similar findings were reported from Ethiopia [36], Burkina Faso and Uganda [37]. In addition, evidence from Bangladesh and Tanzania revealed that breastfeeding counselling increases maternal knowledge on optimal infant feeding [38,39]. Mothers who are knowledgeable on IYCF are more likely to follow the WHO recommendations on practices such as early initiation of breastfeeding, EBF and avoidance of pre-lacteal feeds [40].

Maternal education was not associated with PLF. This was not surprising since most mothers we studied had no formal education. Mothers with no formal education might have had limited access to information on appropriate infant feeding especially through the media, and newspapers. A high level of formal education was reported to be protective against PLF in Eastern Uganda [26].

The findings of this study may have a wide range of implications regarding maternal and infant health, practice and policy on early infant feeding in South Sudan. The high rate of PLF may reduce suckling and production of breastmilk leading to a decrease in EBF [41]. In addition, discarding of colostrum might deny the infant the health benefits leading to an increase in infections and subsequent death. Furthermore, PLF practice disrupts maternal infant bonding which may affect maternal and infant psychology. PLF may also lead to early discontinuation of breastfeeding.

## Strengths and limitations

This was a community-based survey that gave us a glimpse of PLF practice in South Sudan. One limitation of this study was that we interviewed mothers of children aged 0–23 months, regarding infant feeding in the first three days of birth. The responses are likely to be influenced by recall bias, especially among the older infants.We should have over sampled mothers with children aged 0–6 months to shorten the recall period and reduce on recall errors [42]. Secondly, mothers in this study self-reported on PLF practices and this might have led to desirability bias; that is the mothers may have reported the desired answer but not the real practice. Lastly, this study used only a quantitative method. A mixed-methods analysis of PLF practices could have given us in-depth understanding of the factors underlying the practices.

## Conclusion

The prevalence of PLF in South Sudan is high. Predictors of PLF included lack of breastfeeding counselling and discarding of colostrum. Infant feeding counselling should be given to all pregnant women in the health facilities and communities. The counselling should emphasize the health benefits of colostrum and discourage the practice of discarding it.

## Paper context

PLF is an important barrier to EBF. This study was conducted in Jubek State, South Sudan, a country experiencing low intensity conflict. More than half the mothers had given pre-lacteal feeds. Antenatal breastfeeding counselling was associated with a lower risk of PLF, while discarding of colostrum was associated with a higher risk. PLF may further worsen the situation increasing morbidity and mortality. Interventions to mitigate this practice are needed.

## References

[1] United Nations Sustainable development goals. New York: United Nations; 2015 cited 2018 8 30 Available from: http://www.un.org/sustainabledevelopment/summit/

[2] Rollins NC, Bhandari N, Hajeebhoy N, et al Why invest, and what it will take to improve breastfeeding practices? Lancet. 2016;387:491–7. 26869576 10.1016/S0140-6736(15)01044-2

[3] World Health Organization Global strategy for infant and young child feeding: the optimal duration of exclusive breastfeeding. 2001 [cited 2018 8 30]. Available from: http://www.who.int/iris/handle/10665/78801.

[4] Victora CG, Bahl R, Barros AJ, et al Breastfeeding in the 21st century: epidemiology, mechanisms, and lifelong effect. Lancet. 2016;387:475–490. 26869575 10.1016/S0140-6736(15)01024-7

[5] Neovita Study Group Timing of initiation, patterns of breastfeeding, and infant survival: prospective analysis of pooled data from three randomised trials. Lancet Glob Health. 2016;4:e266–e275. 27013313 10.1016/S2214-109X(16)00040-1

[6] Agho KE, Ogeleka P, Ogbo FA, et al Trends and Predictors of prelacteal feeding practices in Nigeria (2003-2013). Nutrients. 2016;8:462. 10.3390/nu8080462 PMC4997375 27483309

[7] Chea N, Asefa A. Prelacteal feeding and associated factors among newborns in rural Sidama, south Ethiopia: a community based cross-sectional survey. Int Breastfeed J. 2018;13:7. 29467812 10.1186/s13006-018-0149-x PMC5819158

[8] United Nations Children’s Fund/World Health Organization Baby-friendly hospital initiative. 2017 [cited 2018 5 23]. Available from: https://www.unicef.org/nutrition/files/BFHI_Case_Studies_FINAL.pdf.

[9] Hajeebhoy N, Nguyen PH, Mannava P, et al Suboptimal breastfeeding practices are associated with infant illness in Vietnam. Int Breastfeed J. 2014;9:12. 25097662 10.1186/1746-4358-9-12 PMC4121620

[10] Edmond KM, Zandoh C, Quigley MA, et al Delayed breastfeeding initiation increases risk of neonatal mortality. Pediatrics. 2006;117:e380–e386. 16510618 10.1542/peds.2005-1496

[11] Legesse M, Demena M, Mesfin F, et al Prelacteal feeding practices and associated factors among mothers of children aged less than 24 months in Raya Kobo district, North Eastern Ethiopia: a cross-sectional study. Int Breastfeed J. 2014;9:189. 25648571 10.1186/s13006-014-0025-2 PMC4308855

[12] Nguyen PH, Keithly SC, Nguyen NT, et al Prelacteal feeding practices in Vietnam: challenges and associated factors. BMC Public Health. 2013;13:932. 24099034 10.1186/1471-2458-13-932 PMC4126174

[13] Berde AS, Yalcin SS, Ozcebe H, et al Determinants of pre-lacteal feeding practices in urban and rural Nigeria; a population-based cross-sectional study using the 2013 Nigeria demographic and health survey data. Afr Health Sci. 2017;17:690–699. 29085396 10.4314/ahs.v17i3.11 PMC5656220

[14] BBC South Sudan becomes an independent nation. 2011 [cited 2018 8 30]. Available from: https://www.bbc.com/news/world-africa-14089843.

[15] Global conflict tracker Civil war in South Sudan - council on foreign relations. 2018 [cited 2018 5 29]. Available from: https://www.cfr.org/interactives/global-conflict-tracker#!/conflict/civil-war-in-south-sudan.

[16] Gezahegn Y, Kassahun W, Dube L Factors associated with acute malnutrition among South Sudanese children in Tierkidi Refugee Camp: a case-control study. Quality in Primary Care. 2017;25:253–258.

[17] United Nations Children’s Fund The state of the world’s children. New York, 2017 [cited 2018 5 28]. Available from: https://www.unicef.org/sowc2017/.

[18] Trish D, Karen O Improving maternal and child health through media in South Sudan 2017 [cited 2018 6 5]. Available from: http://globalhealthstories.com/wp-content/uploads/2017/12/South-Sudan-report-web2.pdf.

[19] South Sudan National Bureau of Statistics Population projections for South Sudan by payam 2015 [cited 2018 6 5]. Available from: http://www.ssnbss.org/sites/default/files/2016-08/population_projections_for_south_sudan_by_payam_2015_2020.pdf.

[20] Dean AG, Sullivan KM, Soe MM Open epi: open source epidemiologic statistics for public health. 2013 [cited 2018 5 20]. Available from: www.OpenEpi.com.

[21] Wamani H, Astrom AN, Peterson S, et al Infant and young child feeding in western Uganda: knowledge, practices and socio-economic correlates. J Trop Pediatr. 2005;51:356–361. 15947011 10.1093/tropej/fmi048

[22] Groppi L, Somigliana E, Pisani V, et al A hospital-centered approach to improve emergency obstetric care in South Sudan. Int J Gynaecol Obstet. 2015;128:58–61. 25270823 10.1016/j.ijgo.2014.07.031

[23] Bennett S, Woods T, Winitha MS, et al A simplified general method for cluster-sample surveys of health in developing countries. 1991 [cited 2018 5 23]. Available from: http://www.who.int/iris/handle/10665/47585. 1949887

[24] World Health Organization Indicators for assessing infant and young child feeding practices. Geneva, 2010 [cited 2018 5 23]. Available from: http://www.who.int/nutrition/publications/infantfeeding/9789241599290/en/.

[25] Tylleskar T, Jackson D, Meda N, et al Exclusive breastfeeding promotion by peer counsellors in sub-Saharan Africa (PROMISE-EBF): a cluster-randomised trial. Lancet. 2011;378:420–427. 21752462 10.1016/S0140-6736(11)60738-1

[26] Engebretsen IM, Wamani H, Karamagi C, et al Low adherence to exclusive breastfeeding in Eastern Uganda: a community-based cross-sectional study comparing dietary recall since birth with 24-hour recall. BMC Pediatr. 2007;7:10. 17331251 10.1186/1471-2431-7-10 PMC1828054

[27] Mgongo M, Mosha MV, Uriyo JG, et al Prevalence and predictors of exclusive breastfeeding among women in Kilimanjaro region, Northern Tanzania: a population based cross-sectional study. Int Breastfeed J. 2013;8:12. 24107593 10.1186/1746-4358-8-12 PMC3852397

[28] Tariku A, Biks GA, Wassie MM, et al Factors associated with prelacteal feeding in the rural population of northwest Ethiopia: a community cross-sectional study. Int Breastfeed J. 2016;11:14. 27231482 10.1186/s13006-016-0074-9 PMC4880979

[29] Jimoh AO, Adaji SE, Adelaiye HA, et al Factors associated with prelacteal feeding practices in a rural northern Nigerian setting. South African Journal of Clinical Nutrition. 2018;31:37–42.

[30] El-Gilany AH, Abdel-Hady DM Newborn first feed and prelacteal feeds in Mansoura, Egypt. Biomed Res Int. 2014;2014:258470. 24895560 10.1155/2014/258470 PMC4033417

[31] Lakati AS, Makokha OA, Binns CW, et al The effect of pre-lacteal feeding on full breastfeeding in Nairobi, Kenya. East African Journal of Public Health. 2010;7:258–262. 21516965 10.4314/eajph.v7i3.64737

[32] Yimer NB, Liben ML Effects of home delivery on colostrum avoidance practices in North Wollo zone, an urban setting, Ethiopia: a cross sectional study. J Health Popul Nutr. 2018;37:4. 29482631 10.1186/s41043-018-0134-4 PMC6389058

[33] Bililign N, Kumsa H, Mulugeta M, et al Factors associated with prelacteal feeding in North Eastern Ethiopia: A community based cross-sectional study. Int Breastfeed J. 2016;11:13. 27190547 10.1186/s13006-016-0073-x PMC4869312

[34] Sreeramareddy CT, Joshi HS, Sreekumaran BV, et al Home delivery and newborn care practices among urban women in western Nepal: a questionnaire survey. BMC Pregnancy Childbirth. 2006;6:27. 16928269 10.1186/1471-2393-6-27 PMC1560161

[35] Legesse M, Demena M, Mesfin F, et al Factors Associated with Colostrum Avoidance Among Mothers of Children Aged less than 24 Months in Raya Kobo district, North-eastern Ethiopia: community-based Cross-sectional Study. J Trop Pediatr. 2015;61:357–363. 26141533 10.1093/tropej/fmv039 PMC4590259

[36] Yenit MK, Genetu H, Tariku A Infant feeding counseling and knowledge are the key determinants of prelacteal feeding among HIV exposed infants attending public hospitals in Ethiopia. Arch Public Health. 2017;75:23. 28536653 10.1186/s13690-017-0191-y PMC5439130

[37] Engebretsen IM, Nankabirwa V, Doherty T, et al Early infant feeding practices in three African countries: the PROMISE-EBF trial promoting exclusive breastfeeding by peer counsellors. Int Breastfeed J. 2014;9:19. 25784955 10.1186/1746-4358-9-19 PMC4362641

[38] Ara G, Khanam M, Papri N, et al Peer counselling improves breastfeeding practices: A cluster randomized controlled trial in urban Bangladesh. Matern Child Nutr. 2018;14:e12605. 29660858 10.1111/mcn.12605 PMC6055706

[39] Hashim TH, Mgongo M, Katanga J, et al Predictors of appropriate breastfeeding knowledge among pregnant women in Moshi Urban, Tanzania: a cross-sectional study. Int Breastfeed J. 2016;12:11. 28228840 10.1186/s13006-017-0102-4 PMC5307776

[40] Bica OC, Giugliani ER Influence of counseling sessions on the prevalence of breastfeeding in the first year of life: a randomized clinical trial with adolescent mothers and grandmothers. Birth. 2014;41:39–45. 24654636 10.1111/birt.12097

[41] Rogers NL, Abdi J, Moore D, et al Colostrum avoidance, prelacteal feeding and late breast-feeding initiation in rural Northern Ethiopia. Public Health Nutr. 2011;14:2029–2036. 21557873 10.1017/S1368980011000073

[42] Greiner T Exclusive breastfeeding: measurement and indicators. Int Breastfeed J. 2014;9:18. 25349624 10.1186/1746-4358-9-18 PMC4209171

